# Community evolution on Stack Overflow

**DOI:** 10.1371/journal.pone.0253010

**Published:** 2021-06-17

**Authors:** Iraklis Moutidis, Hywel T. P. Williams

**Affiliations:** 1 Computer Science, University of Exeter, Exeter, United Kingdom; 2 Alan Turing Institute, London, United Kingdom; University of Pisa, ITALY

## Abstract

Question and answer (Q&A) websites are a medium where people can communicate and help each other. Stack Overflow is one of the most popular Q&A websites about programming, where millions of developers seek help or provide valuable assistance. Activity on the Stack Overflow website is moderated by the user community, utilizing a voting system to promote high quality content. The website was created on 2008 and has accumulated a large amount of crowd wisdom about the software development industry. Here we analyse this data to examine trends in the grouping of technologies and their users into different sub-communities. In our work we analysed all questions, answers, votes and tags from Stack Overflow between 2008 and 2020. We generated a series of user-technology interaction graphs and applied community detection algorithms to identify the biggest user communities for each year, to examine which technologies those communities incorporate, how they are interconnected and how they evolve through time. The biggest and most persistent communities were related to web development. In general, there is little movement between communities; users tend to either stay within the same community or not acquire any score at all. Community evolution reveals the popularity of different programming languages and frameworks on Stack Overflow over time. These findings give insight into the user community on Stack Overflow and reveal long-term trends on the software development industry.

## Introduction

The software and computing industries change rapidly as new technologies and platforms are introduced. The ecosystem of programming languages, methodologies and developers is highly complex, with tools and behaviours continually adapting to internal and external forces. Widespread adoption of web-based and/or ‘cloud’ computing paradigms has altered not only the software itself (in terms of languages and architectures) but also how software is created. The sharing and re-use of code via platforms such as GitHub(https://github.com/), Bitbucket (https://bitbucket.org), and Source Forge (https://sourceforge.net/) enable more effective collaboration, while the easy availability of programming expertise online on sites such as Stack Overflow (https://stackoverflow.com/) and Quora (https://www.quora.com) has changed how individuals seek help with programming challenges. Whereas once an individual might seek an answer in a text book or from an office colleague, it has become the norm to seek help online.

Question-and-answer websites such as Stack Overflow are widely used by programmers and researchers, forming a large repository of valuable knowledge related to the software development, computing, and data science industries. Software developers rely on such websites to acquire knowledge, solve problems, seek snippets of code for reuse, improve their own code, and discuss technical concepts. Stack Overflow also helps individuals gain visibility to establish professional standing and reputation. At time of writing, the Stack Overflow community has more than 14 million registered users who have asked 21 million questions and received 31 million answers; many more unregistered users utilise the publicly displayed questions/answers provided by others without contributing new content. Approximately 70% of questions receive an answer (https://stackexchange.com/sites#technology) making the platform one of the best places to seek help or discuss emerging technology issues.

Stack Overflow is written by many and read by many. To help ensure good content is easily visible, Stack Overflow provides a voting system where users that contribute high quality answers or interesting questions are assigned positive votes and thematic tags (e.g. ‘C++’, ‘Python’, ‘Machine-Learning’) by other users. Thereby a ‘reputation’ score is formed that identifies the most knowledgeable users for specific fields. The reputation score is also used to give privileged roles to high-ranked users, such as up-voting, editing and moderating the community. Moderation of the community is quite strict; only well-documented, on-topic and correctly tagged questions/answers are accepted. These features ensure that content is of high quality, but also provide a rich resource for social analysis of the platform.

The social dimensions to online programming platforms such as GitHub and Stack Overflow are an intrinsic part of their function. Various studies have tried to understand social factors, for example, seeking to identify influential users [1, 2] or understand their general properties as socio-technical systems [3]. The social component also provides useful information about wider trends in the software industry, with user activity reflecting the shifting popularity of different technologies.

Here we analyse the evolution of the Stack Overflow user community over a relatively long period (2008-2020). By tracking the usage of different tags by individual users, we are able to provide insight into the clusters of topics that are the focus of clusters of users, and observe trends in the adoption of new programming languages and technologies. It is reasonable to assume that trends on Stack Overflow, revealed by analysis of users, tags and various platform metrics, are reflective of wider trends in the software industry. Thus we use Stack Overflow as a lens with which to study attention to different technologies, reveal technology clusters defined by the user groups that utilise them, and observe the movement of people between different technological clusters over time.

The core of our methodology is the construction of networks that link users to each other based on the tags that define their (shared) expertise. Within these networks we use community detection algorithms to identify sub-communities representing groups of users focused on particular technology clusters, using the set of tags associated with users to characterise each sub-community. By analysing a temporal sequence of such networks we are able to explore the concurrent evolution of the programming community and underpinning technologies over time. We examine how the various sub-communities relate to each other and identify different technologies with common applications. By monitoring the movement of users between communities over time, we show that the majority of users either remain in the same community or didn’t acquire any score, and that only a small fraction of users migrate between communities. The rise and fall of different technologies, revealed by the number of users who are interested in them and the way technologies are clustered, provides insight into the dynamics of the tech industry during a period of rapid change.

The next section describes some relevant Background, including a brief description of the operation of the Stack Overflow platform and some related work using similar data. In the Dataset & Methods section, we describe the data collection and pre-processing steps, as well as the processes used to generate the findings given in the Results section. The Discussion section concludes the paper.

## Background

### Stack Overflow

Stack Overflow is a self-moderating online Question & Answer forum. Stack Overflow questions are generally hard, requiring expertise and domain knowledge to provide a good answer.

Stack Overflow’s success is largely due to the engaged and active user community that collaboratively manages the site. Content is heavily curated by the community. Duplicate or similar questions are quickly identified as such and merged with existing questions. Posts (questions or answers) that are unhelpful or irrelevant are removed. As a result of this self-regulation, content on Stack Overflow tends to be of high quality.

The quality of each post is collaboratively evaluated using a voting system. Each question or answer can receive up-votes or down-votes from users, with the sum of votes (up-votes minus down-votes) acting as its overall voting score. The votes awarded to a user’s posts is accumulated in their ‘reputation’, another type of score associated with individual users and intended to identify expert users.

Reputation brings moderation privileges. Each user gets the ability to up-vote a post when their reputation score reaches 15 points and the ability to down-vote a post when their reputation reaches 125 points. On Table 1 we present all the available privileges on the platform as well as the required reputation for each one and the percentage of the total users that poses them.

**Table 1:** Users distribution by Stack Overflow privileges.

Reputation	Privilege	Description	Percentage
25000	Access to site analytics	Access to internal and Google site analytics	0.05%
20000	Trusted users	Expanded editing, deletion and undeletion privileges	0.07%
15000	Protect questions	Mark questions as protected	0.1%
10000	Access to moderator tools	Access reports, delete questions, review reviews	0.16%
5000	Approve tag wiki edits	Approve edits to tag wikis made by regular users	0.35%
3000	Cast close and reopen votes	Help decide whether posts are off-topic or duplicates	0.6%
2500	Create tag synonyms	Decide which tags have the same meaning as others	0.71%
2000	Edit questions and answers	Edits to any question or answer are applied immediately	0.88%
1500	Create tags	Add new tags to the site	1.14%
1000	Established user	You’ve been for a while; see vote counts	1.61%
1000	Create gallery chat rooms	Create chat rooms where only specific users may talk	1.61%
500	Access review queues	Access first posts and late answers review queues	2.81%
250	View close votes	View and cast close/reopen votes on your own questions	4.37%
200	Reduce adds	Some add are automatically disabled	4.77%
125	Vote down	Indicate when questions and answers are not useful	6.42%
100	Edit community wiki	Collaborate on the editing and improvement of wiki posts	7.62%
100	Create chat rooms	Create new chat rooms	7.62%
75	Set bounties	Offer some of your reputation as bounty for a question	8.9%
50	Comment everywhere	Leave comments on other people’s posts	11.17%
20	Talk in chat	Participate in the site’s chat rooms	18.49%
15	Flag posts	Bring content to the attention of the community via flags	19.44%
15	Vote up	Indicate when questions and answers are usefull	19.44%
10	Remove new user restrictions	Post more links, answer protected questions	25.28%
10	Create wiki posts	Create answers that can be easily edited by most users	25.28%
5	Participate in meta	Discuss the site itself; bugs, feedback, and governance	26.20%
1	Create posts	Ask a question or contribute an answer	100%

Percentage of users possessing privileges of the Stack Overflow website. Every user poses all the privileges that require less or equal reputation than he/she currently has. More information can be found here: https://stackoverflow.com/help/privileges

Another key mechanism on the Stack Overflow site is the use of tags to identify the content or theme of each post. When a user asks a question, the platform prompts them to add a small number of content tags (at least one and at most five).

A user can refer to the website’s help center (https://stackoverflow.com/help/) where he can learn more about Stack Overflow and its rules.

### Related work

The research literature includes various studies that analyse question-and-answer websites, typically using data from Stack Overflow, Quora (https://www.quora.com/) or Yahoo! Answers (https://answers.yahoo.com/). These studies can be loosely grouped into three categories: studies of network structure, studies of content, and studies of information retrieval. We cover these in turn below.

Studies of network structure explore the relationships between entities, such as users, posts or tags, that are associated with question-and-answer websites. Communities can be identified from network structure and analysed to detect key actors, as well as the main interests and typical behaviours of the users. Silvestri et al [4] describe a methodology for linking user accounts between platforms (across Stack Overflow, Github and Twitter) based on user attributes and platform specific services, examining different account matching strategies. Wang, Liu & Fan [5] introduce a methodology to discover similar users in online communities based on the tags they share. Beyer and Pinzger [6] introduce an approach to group tag synonyms to meaningful topics by creating networks, and investigating community detection algorithms to build meaningful groups of tags. Rosen & Shihab [7] analyze more than 13 million posts from Stack Overflow to examine topics of discussion amongst mobile application developers, finding that this community is focused on app distribution, mobile APIs, data management, sensors/context, mobile tools, and user interface development. Fu, Yu & Benson [8] analyze the tag networks of several Stack Exchange communities and develop a simple generative model that creates random bipartite graphs of tags and questions. Halavais et al [9] investigate whether individual badge-seeking behaviour is motivated by exposure to others’ achievements, concluding that ‘general’ badges are closely related to tenure on the site, while more numerous ‘tag’ badges are associated with socially influenced differentiation. Papoutsoglou, Kapitsaki & Angelis [10] introduce a methodology for modeling the effect of the badge reward system on the personal traits of Stack Overflow users based on data recorded before and after the award time, employing the ‘Big Five’ personality Factors.

Studies of content on online question-and-answer communities typically analyse the content and metadata of questions and answers. Calefato et al [11] investigate how Stack Overflow users can increase the chance of getting their answer accepted when writing an answer or making comments, finding that information presentation, timing and affect all have an impact on the success of a post. Schenk & Lungu [12] use the geospatial metadata associated with each Stack Overflow user to understand how different geographic regions contribute to the knowledge market represented by the community. They find that Europe and North America are the principal (and roughly equal) contributors, with Asia as a distant third (mainly India), followed by Oceania, which even in fourth position still contributes more than South America and Africa combined. Morrison & Murphy-Hill [13] aim to support career planning and staff development by identifying age-related trends in Stack Overflow data, observing that user reputation scores increase with age well into their fifties, that programmers in their thirties tend to focus on fewer areas than those younger or older in age, and that there is not a strong correlation between age and reputation scores for any specific knowledge areas. Ragkhitwetsagul et al [14] investigate the quality of code Stack Overflow answers contain, how often it gets adopted (cloned) by the community, and how often it is reviewed/modified by the answer author. Their research shows that a significant amount of reused Stack Overflow code (∼66%) is outdated, of which (∼6.6%) was buggy and harmful for reuse. Vasilescu et al. [15] similarly investigate the relation between Stack Overflow questions and answers, and the software development process, as reflected by code changes committed to the GitHub code repository. They find that Stack Overflow activity rates correlate with activity in GitHub.

Studies of information retrieval related to question-and-answer communities typically adopt a perspective where a question is viewed as a ‘query’ and answers as ‘results’. De Sousa, Campos & Maia [16] make use of ‘crowd knowledge’ from Stack Overflow to recommend information to developers. Ranked lists of question/answer pairs are recommended in response to a query based on textual similarity and the question/answer vote scores. Liu et al [17] predict ‘searcher satisfaction’ by analysis of a large number of web searches that result in a visit to a popular question-and-answer site (Yahoo! Answers). They identify query clarity, query-to-question match, and answer quality, as key factors in predicting searcher satisfaction. Xu et al [18] use Stack Overflow as their source of question-and-answer threads, achieving good results with an attention-based model that predicts which answer will be preferred by the user posting the original question. Zhang et al [1] propose a methodology for duplicate question detection in question-and-answer communities, adopting a classification approach based on text vectorisation and neural networks.

## Data collection & methods

Our methodology uses interactions between users and tags on Stack Overflow to explore trends in software development and technology usage over time. The assumption is that the tags attached to posts by a user, and the reputation score they acquire from posts using those tags, form a profile for each user that defines their interests and expertise. These profiles can be used to link pairs of users based on the similarity of their expertise. Pairwise links can be aggregated to form a network of users within which community structure reflects groupings of users and technologies. These networks can be studied over time to explore trends.

This section describes how the data were collected and some pre-processing steps that were used to, first, associate content tags to ‘answer’ posts (this information is not provided in the data files), and second, assign scores to each user based on the tags they used in a given time period. Then we describe the main parts of the network creation and analysis methods, including how each community was characterised based on its dominant tags. Overall, this analysis pipeline permits the Stack Overflow developer community to be studied over time and to thereby reveal trends in software development and technology usage. The source code of this research can be found here https://github.com/imoutidi/stackoverflow.

### Data collection and pre-processing

For our analysis we obtained all questions, answers (https://archive.org/download/stackexchange/stackoverflow.com-Posts.7z), votes (https://archive.org/download/stackexchange/stackoverflow.com-Votes.7z) and tags (https://archive.org/download/stackexchange/stackoverflow.com-Tags.7z) on the Stack Overflow website between its inception on 31st July 2008 and 31st December 2020. All the data were retrieved from the Archive.org platform (https://archive.org/download/stackexchange), which hosts the entire history of every Stack Exchange community, including the tags used to annotate questions and the votes of each question and answer. This is an anonymized dump of all user-contributed content on the Stack Exchange network. Each site is formatted as a separate archive consisting of XML files zipped via 7-zip using bzip2 compression and updated every three months. Each site archive includes Posts, Users, Votes, Comments, PostHistory and PostLinks.

All user content contributed to the Stack Exchange network is cc-by-sa 4.0 (https://creativecommons.org/licenses/by-sa/4.0/) licensed, intended to be shared and remixed. The acquisition, processing and presentation of these data fully complies with the terms and conditions of the Stack Exchange network.

The XML files were quite large. The *posts* file was ∼70 gigabytes, the *votes* file was ∼16.7 gigabytes, and the *tags* file smaller at ∼5 megabytes. From the creation of the platform on 31st July 2008 until December 2020 there were ∼46 million posts, of which ∼18 million were questions and ∼27 million were answers.

The Stack Overflow platform awards reputation scores depending the vote type (https://stackoverflow.com/help/whats-reputation). If an answer gets accepted the author of the question gets 5 score points and the author of the answer 15 points. If a question gets up-voted the author gets 10 points and if an answer gets up-voted the author gets 10 points. If a post gets down-voted the author gets -2 points, as also does the user who cast the down-vote.

The first step of our analysis was to parse the data files to group together posts that were created in each month. For some analyses, we used a monthly time unit, for others yearly, making a 1-month bin size suitable as input for both.

Before user networks could be created, we first associated each user with a set of tags reflecting their interests and expertise. The up-votes each user receives on a post are also associated with the tags assigned to the post. For example if a user receives 100 reputation on a post the co-responding tags will be assigned to the user as well and each one of them will receive the same reputation. This creates a ranking of related tags for each user. Our goal is to utilise those tags to form relations between users based on how similar their tags are. Those tags are based on the reputation score the users acquired from posts containing those tags.

The data archive provides the list of tags associated with each question post, but does not provide this information for answer posts. Therefore, we annotated each answer post with the same tags as its parent question post, using the *ParentId* attribute to recover the corresponding question record. To do that efficiently with the large files, we created indexes for each question post pointing to the time period the question was posted, then used these to load only the questions of each time period, indexed on their post *Id*, then retrieved each question’s tags and added them to an expanded record for each answer post.

At the end of processing, the product was a data dictionary for each month with the user IDs as keys and a ranked list of their top tags based on the reputation score they acquired.

### Graph creation and community detection

For our analysis, we created graphs where the nodes are the users and the edges between them represent how related two users are in their technology focus, based on how similar tags those users have. For calculating the weight of each edge we first normalized the score of each user’s tags creating a vector where the sum of all tag scores is 1 and then applied the Manhattan distance between the user’s vectors (Eq 1). This way we get 0 for identical users and 2 for users with completely different tags. We convert the distance into similarity by subtracting it from two. Our similarity metric determines which tags mostly represent each user and by comparing the users vectors we can calculate how similar two users are.

$$\begin{array}{c} \;\;EdgeWeight\left(U_{1},U_{2}\right)=2-\sum_{t\in Tags}\left|ScoreU_{1}\left(t\right)-ScoreU_{2}\left(t\right)\right| \end{array}$$ (1)

Where *U*_1_, *U*_2_ are the users and *Tags* the union of their tags.

The motivation behind this graph creation approach is to link users that have similar tags. Using this similarity-based edge creation approach we cluster users that have similar technology interests. Alternatively, we might have linked users that interacted directly on the StackOverflow platform, e.g. by providing an answer to a query. This kind of user interaction is subsumed within our similarity-based edges, since both questions and answers are associated with the same tags. Our approach additionally allows users with similar interests to be linked, even if they have never interacted directly; this aspect serves to provide a more coherent network structure. We then monitor if our methodology produces a meaningful structure, detect user communities and annotate them based on the tags of the users belonging to each community. Our priority here is to reveal which tags are grouped together on each community and, by annotating each community, to understand which technologies are used on each software development field.

The approach we took for dynamic community discovery was the Instant Optimal Community Detection approach [19] which considers that the detected communities on every time step (one year in our case) are independent. This approach consists of two stages. The Identify stage where communities are detected on each step of the evolution and the Match stage where the detected communities on each step are aligned with the communities of the other steps. For each calendar year in the period 2008- 2020, we created one graph containing the top 100 thousand users based on their reputation that were active during that year to sparsify the networks and their tag-based relationships (For years 2008 (∼17000 users) and 2009 (∼65000 users) we included all active users because Stack Overflow launched on 31st July 2008 and got bigger after 2009).

We then applied the InfoMap [20] community detection algorithm to identify community structure within these graphs. Infomap is a pattern-based community detection algorithm and is based on the concept of patterns of random movement (walks) among the nodes of a network. The main intuition of this method is that a community can be defined as a group of nodes where a random walker is more likely to be trapped in. This concept can be treated as an increased flow circulation pattern between the nodes of the same community. We chose this algorithm based on its performance on networks with sizes around 100 thousand nodes [21–23]. We used the implementation of Edler, Eriksson and, Rosvall which can be found here: https://mapequation.github.io/infomap/.

### Community identification and persistence over time

To characterise each community of users, we utilised their reputation scores on the tags that were used to create its edges. This was done by summing the scores for tags shared by the users within each community (i.e. that were used to create the graph edges adjacent to that community). The summed scores were used to create a ranking of tags based on the total associated reputation score acquired by the community users. Using the top tags of each community ranking we are able to characterise the community by its core topics and technologies, and identify the part of the software industry that it represents.

For each year, we detected a number of communities in the user-tag graph. Characterisation using tags suggested that many of the bigger communities were present in almost every year, while smaller communities tended to be more volatile, appearing and disappearing. For the bigger communities we wanted to investigate their consistency in terms of the users that constitute them in each year. Our intuition is that a persistent community should appear as a pair of communities in consecutive years that have a large number of users in common and a low number of users in common with any other community. For each community in each year, we calculated the percentage of users in common with each of the previous year’s communities, using the percentage overlap between the most-similar communities as a measure of their persistence. Persistence was also assessed qualitatively by the similarity of dominant tags over time.

## Results

### Usage of Stack Overflow over time

**Fig 1:**
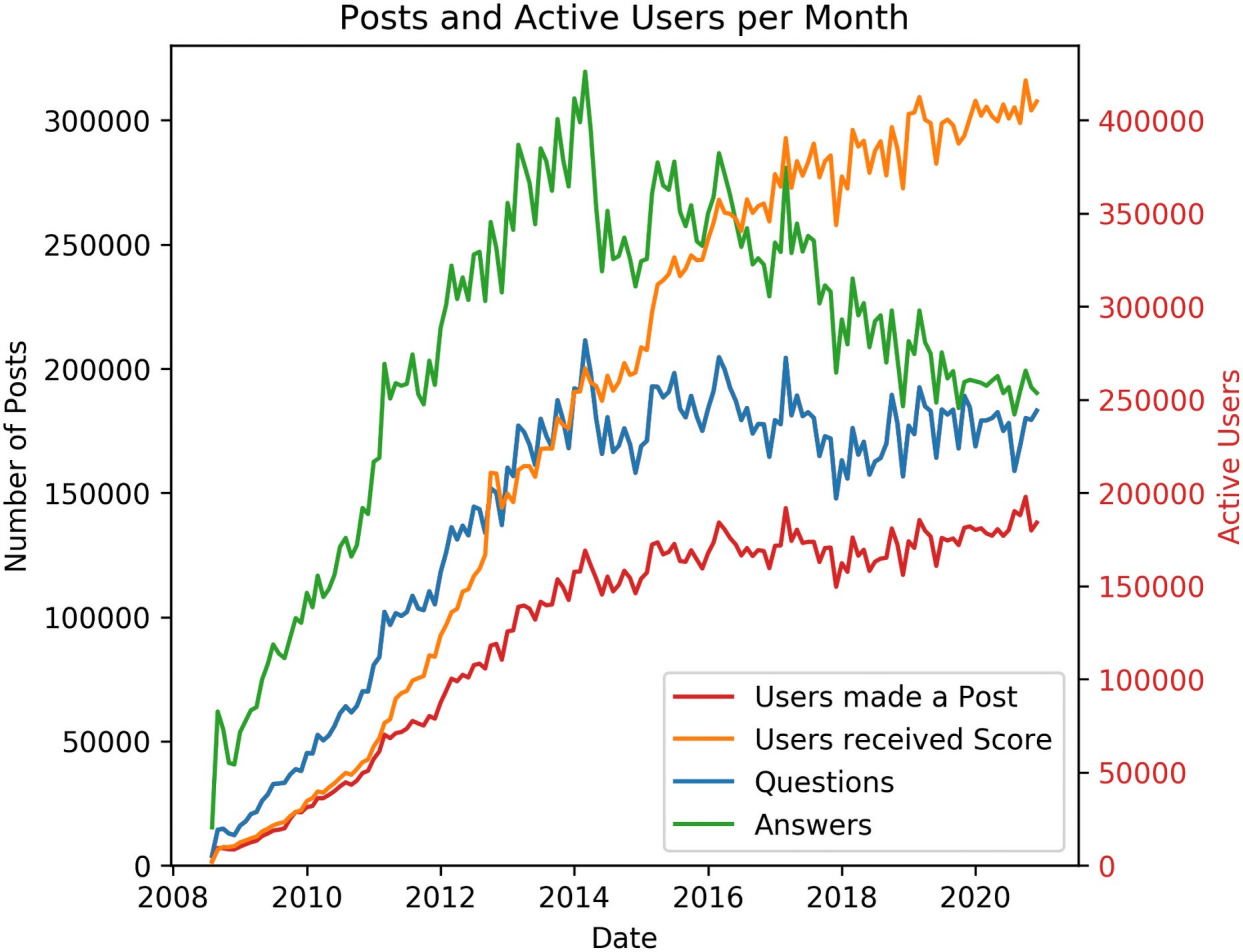
Number of posts and active users on Stack Overflow in each month from August 2008 to December 2020. Plot displays the number of questions and answers posted (left axis), and the number of users creating posts or receiving score via up-votes (right axis).

Fig 1 displays the number of questions and answers posted on Stack Overflow for every month since August 2008 until December 2020, as well as the number of active users (defined as those users that created a post—question or answer—or received scores from up-votes on a historic post). The amount of posts increased from 2008 until 2014, with more answers than questions (perhaps to be expected since each question can have more than one answer). We observe a significant drop in the number of answers and questions posted after 2014. The number of active users making posts stabilises after 2015 while the number of users receiving up-votes continues to increase. During the same period, there is a decrease in the number of answers, while the number of questions reaches an equilibrium.

The reasons behind the drop in activity after 2014 are out of the scope of our work, but a possible reason is the creation at that time of the Stack Overflow Meta community (https://meta.stackoverflow.com). Stack Overflow Meta is a forum where users discuss the workings and policies of Stack Overflow, rather than discussing programming itself. It is separated from the main question-and-answer site to reduce noise, while providing a legitimate space for people to ask how and why the Stack Overflow site works the way it does. A lot of questions more suitable for the Meta community were moved away from the main platform by the moderators, giving a plausible explanation for the significant drop in 2014.

There is a discussion on the Meta community (https://meta.stackoverflow.com/questions/256003/on-large-communities-decaying-over-time-being-nice-or-mean-and-stack-overflow/256084#256084) about the changes in user activity and what this means for the operation of Stack Overflow; we encourage the reader to refer to it. What is probably happening is that the community can handle a specific amount of questions each month. If the number of questions exceeds a threshold (about 180 thousand questions) the community gets saturated and many questions are not answered. This discourages users, especially new ones, from asking new questions and answer on other peoples questions, resulting on a decrease in answers and stable amount of questions. The amount of users creating new posts is increasing until 2014 and then it seems to stabilize on around 180 thousand users. The amount of users receiving score is increasing since 2008 and reaches about 400 thousand users. This means that older posts are receiving votes from the users thus posts can be useful to the community for a long period of time, even tho the software development industry is rapidly evolving and changing.

### Popularity of tools and technologies

To observe trends in the popularity of different tools and technologies in the software industry, we tracked the accumulated monthly votes given by Stack Overflow users to posts labelled with three categories of tags. Each time a question or answer received a vote, we incremented the score for every tag associated with the question. Scores for each tag were aggregated over monthly time periods. These scores show which tags (and by extension, which topics or technologies) are getting positive attention from the software development community.

**Fig 2:**
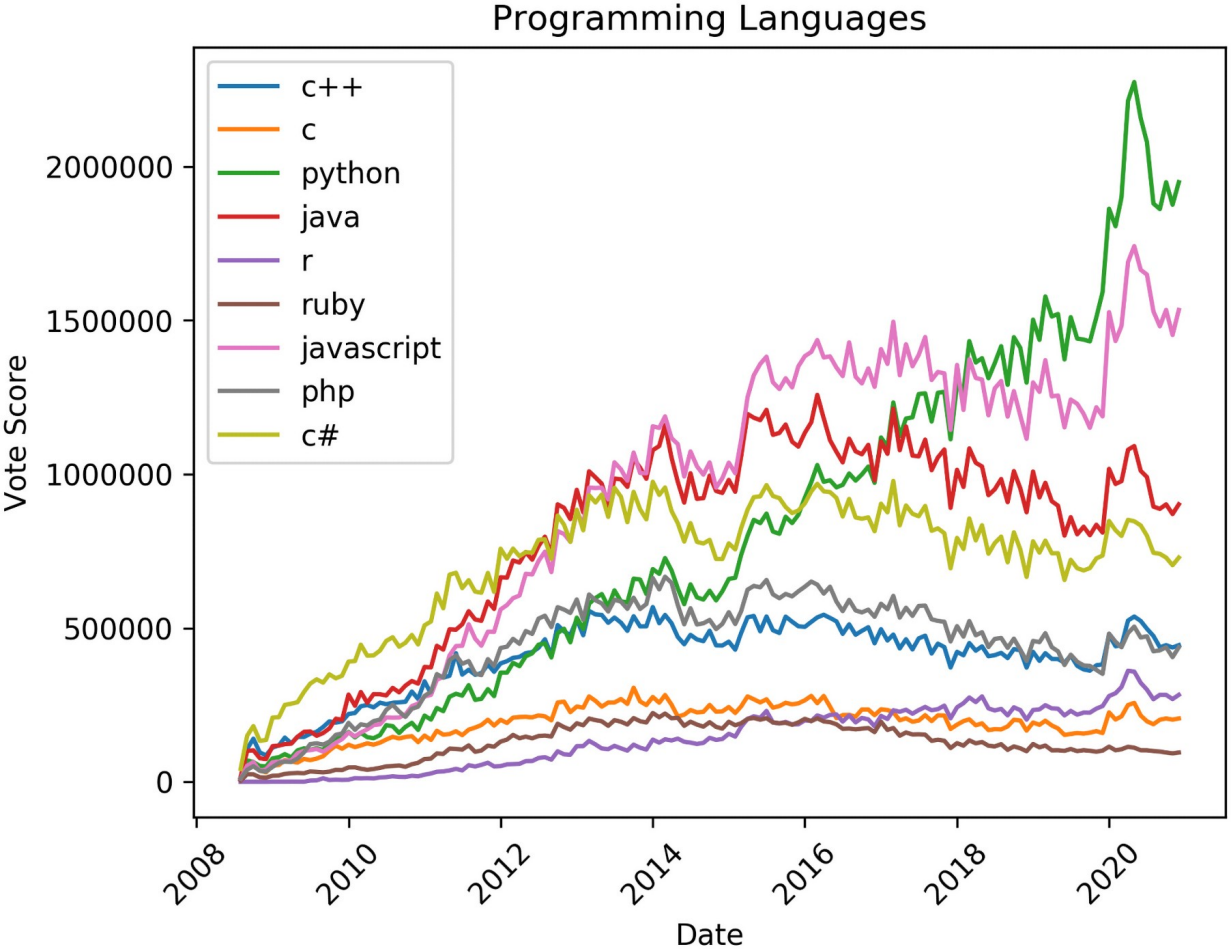
Trends in tag popularity: Programming languages.

Fig 2 shows tags representing popular programming languages. Trends show some similar patterns to those for overall numbers of posts (see above), with almost every tag showing a drop in attention from 2014, and most languages showing decreasing interest after 2017, similar to the number of answers (see Fig 1). However, the trends of languages relative to each other are interesting. The only languages that consistently increase are Python and R, perhaps reflecting the adoption of these languages for data analysis. Python becomes the top-ranked language by the end of 2020, after growing significantly to replace Javascript.

**Fig 3:**
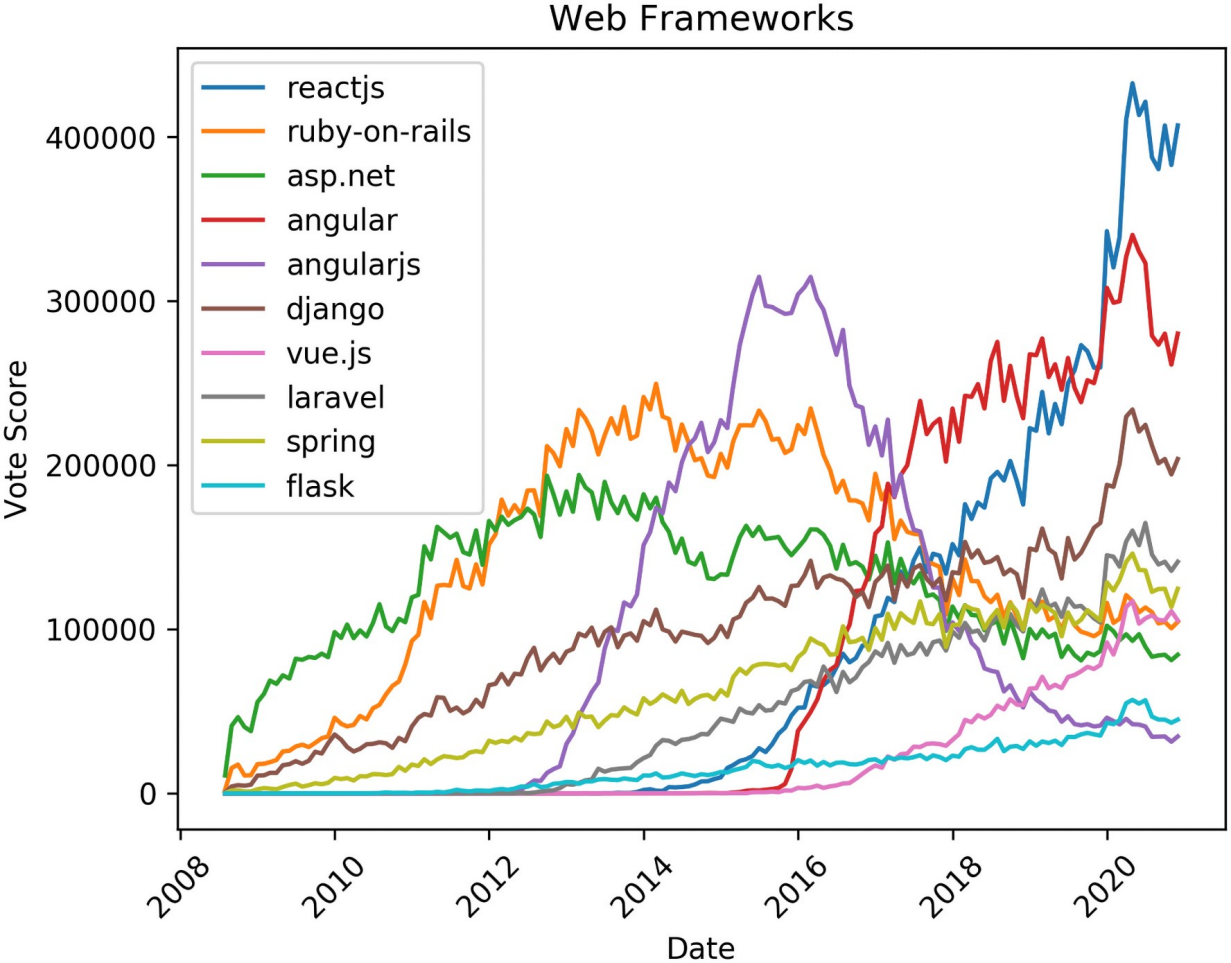
Trends in tag popularity: Web programming frameworks.

Fig 3 shows tags representing popular web programming frameworks. An interesting observation is the rise of AngularJS between 2012 and 2016, then its subsequent replacement by Angular. These two frameworks are open-source JavaScript frameworks and Angular is considered a direct alternative to AngularJS; this analysis suggests that Angular is replacing AngularJS for web development. Another framework that is rapidly growing is ReactJS. Well-known web frameworks like ASP.NET and Ruby-on-Rails seem to decline, while Django and Spring steadily increase since the beginning of the Stack Overflow platform.

**Fig 4:**
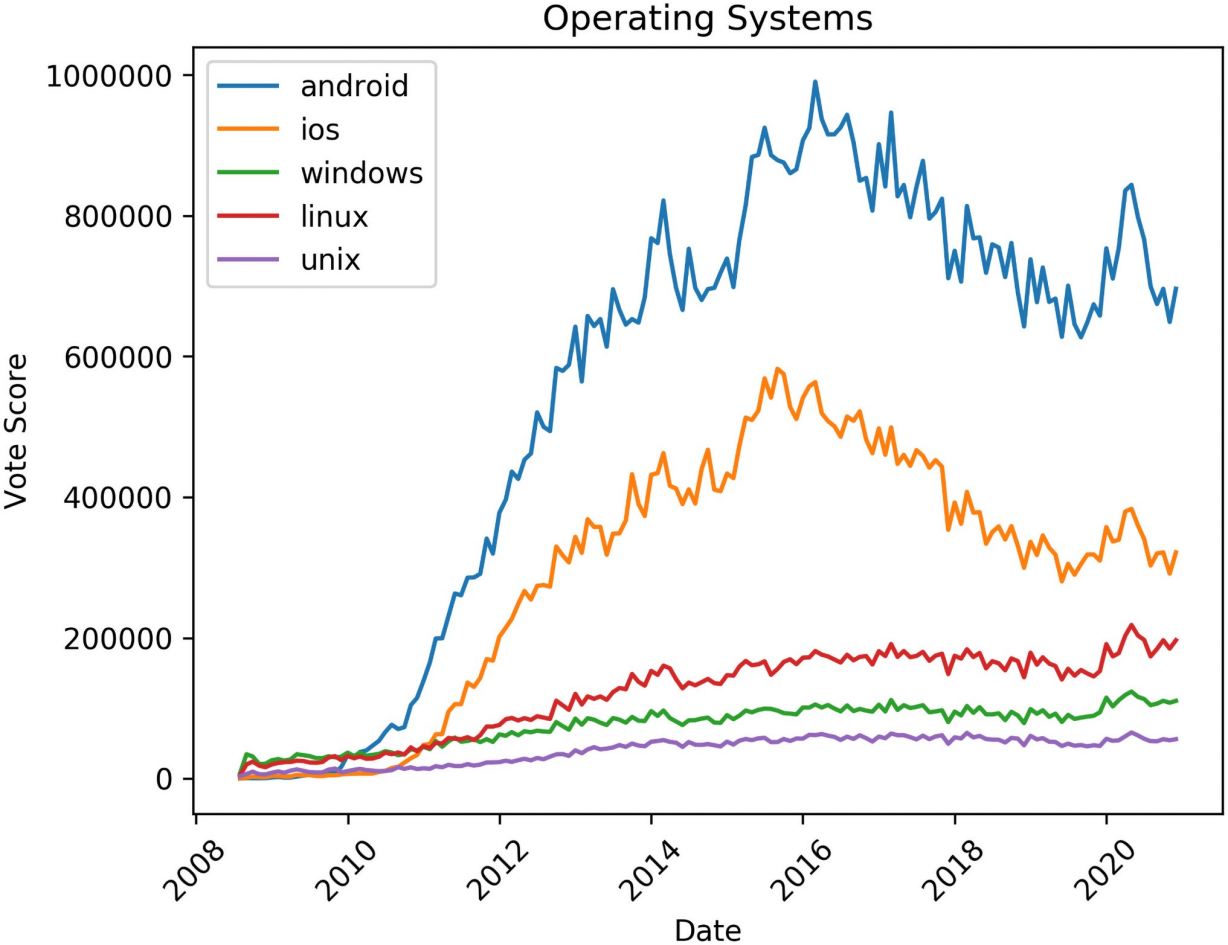
Trends in tag popularity: Operating systems.

Fig 4 shows tags relating to operating systems. The most dominant tag is Android followed by iOS, showing a bias to discussion of programming for mobile platforms. Surprisingly the Linux tag receives more attention than the Windows tag, suggesting that although the majority of personal computers in the world run the Windows operating system, the community of software developers on Stack Overflow is more interested in Linux.

### User communities on Stack Overflow

**Fig 5:**
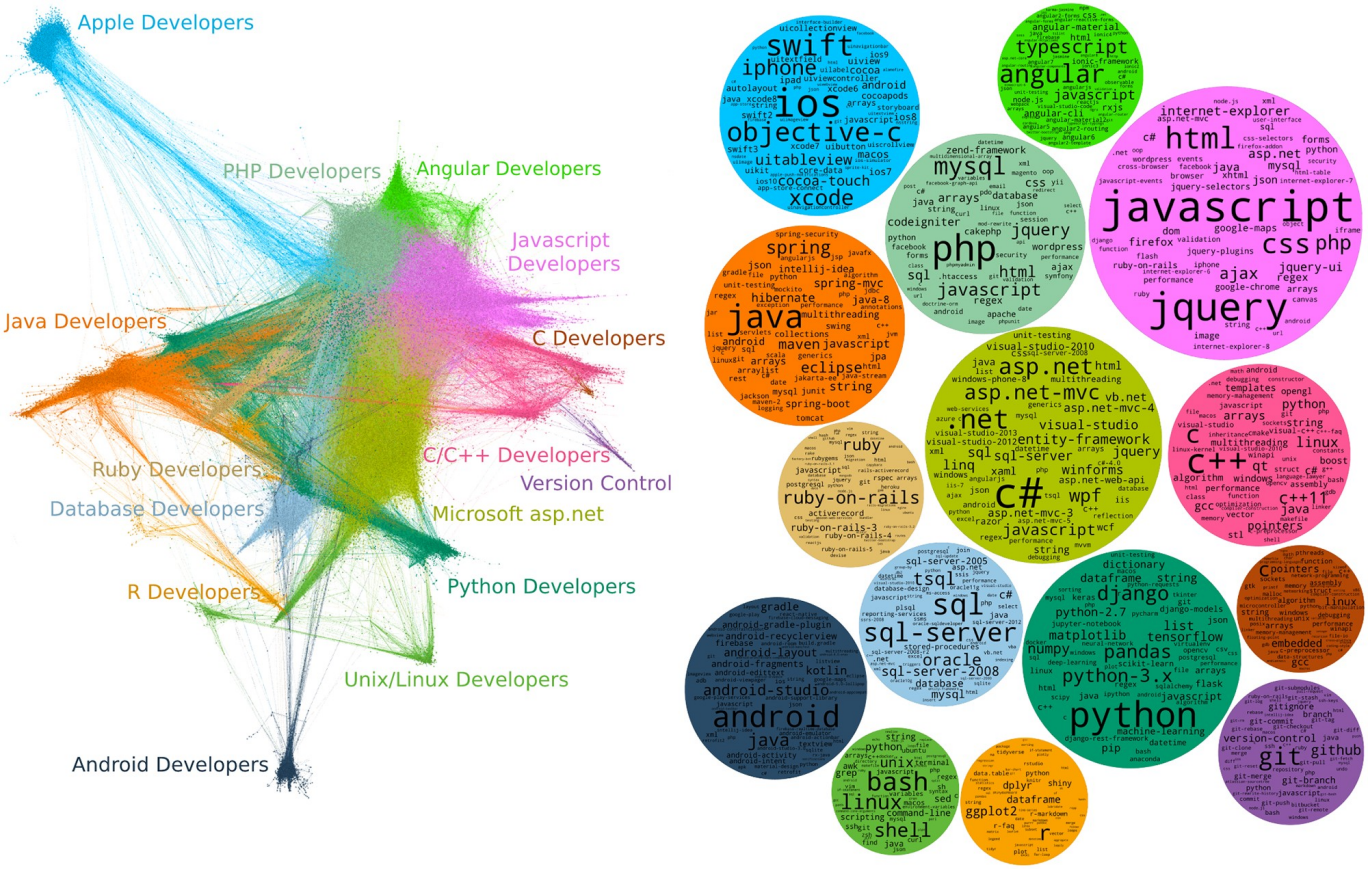
Stack Overflow community graph. User-tag interaction graph created from all available data between 2008 and 2020. Left: Communities of users within the Stack Overflow platform. Right: Tag clouds corresponding to each community showing the most frequent tags used by the users in that community.

Initially we created a graph using all the available data from 2008 until 2020. This graph is shown in Fig 5 (left), which displays the nodes/edges using the ForceAtlas2 continuous graph layout algorithm [24] from the Gephi visualization tool. Community detection was used to identify the 18 largest communities formed by Stack Overflow users, based on all interactions between 2008 and 2020; these are shown by node colours and annotated with a community label derived from their dominant tags. Fig 5 (right) shows the most frequent tags associated with posts by the users forming each community. Here each community is represented by a circle, using the same colour scheme as the left-hand plot for comparison, and sized proportionally to its number of members. Within each community (circle) the dominant tags are presented with size proportional to their usage. Table 2 displays network metrics for the detected communities. We can see that almost all communities have low density and about 6 of them have modularity higher than 0.4. this is a good indicator that those communities could be further split into sub-communities. we will further investigate this on future work.

**Table 2:** Network metrics of the detected communities.

Community Name	Density	Modularity	Avg. Degree	Avg. Weighted Degree
Android Developers	0.01	0.383	22.4	284.9%
Angular Developers	0.01	0.263	28.7	356.5%
Apple Developers	0.006	0.41	23.4	289.5%
C Developers	0.004	0.392	6.1	76.3%
C/C++ Developers	0.003	0.681	9.16	114.2%
Database Developers	0.019	0.322	53.2	680.8%
Java Developers	0.009	0.425	27.1	340.7%
Javascript Developers	0.006	0.351	47.4	590.2%
Microsoft asp.net	0.007	0.345	61.3	762.6%
PHP Developers	0.014	0.21	95.8	1195.6%
Python Developers	0.005	0.61	19.2	227.7%
R Developers	0.005	0.421	9.5	117.2%
Ruby Developers	0.01	0.304	19.9	247.3%
Unix/Linux Developers	0.004	0.736	7.9	99.2%
Version Control	0.005	0.411	6.8	79.5%

The detected communities correspond to identifiable sub-fields of the software development industry, such as web developers or Android developers. The plots provide insights about the number of developers working in a specific sub-field, as well as the specific technologies that are popular in each sub-field. For example, some of the top tags of the Android community are android-layout, android-activity, android-intent, android-studio and sqlite. This provides information about which components, tools, IDEs and database engines are discussed on Stack Overflow in the context of the Android platform.

### Studying user communities over time

**Fig 6:**
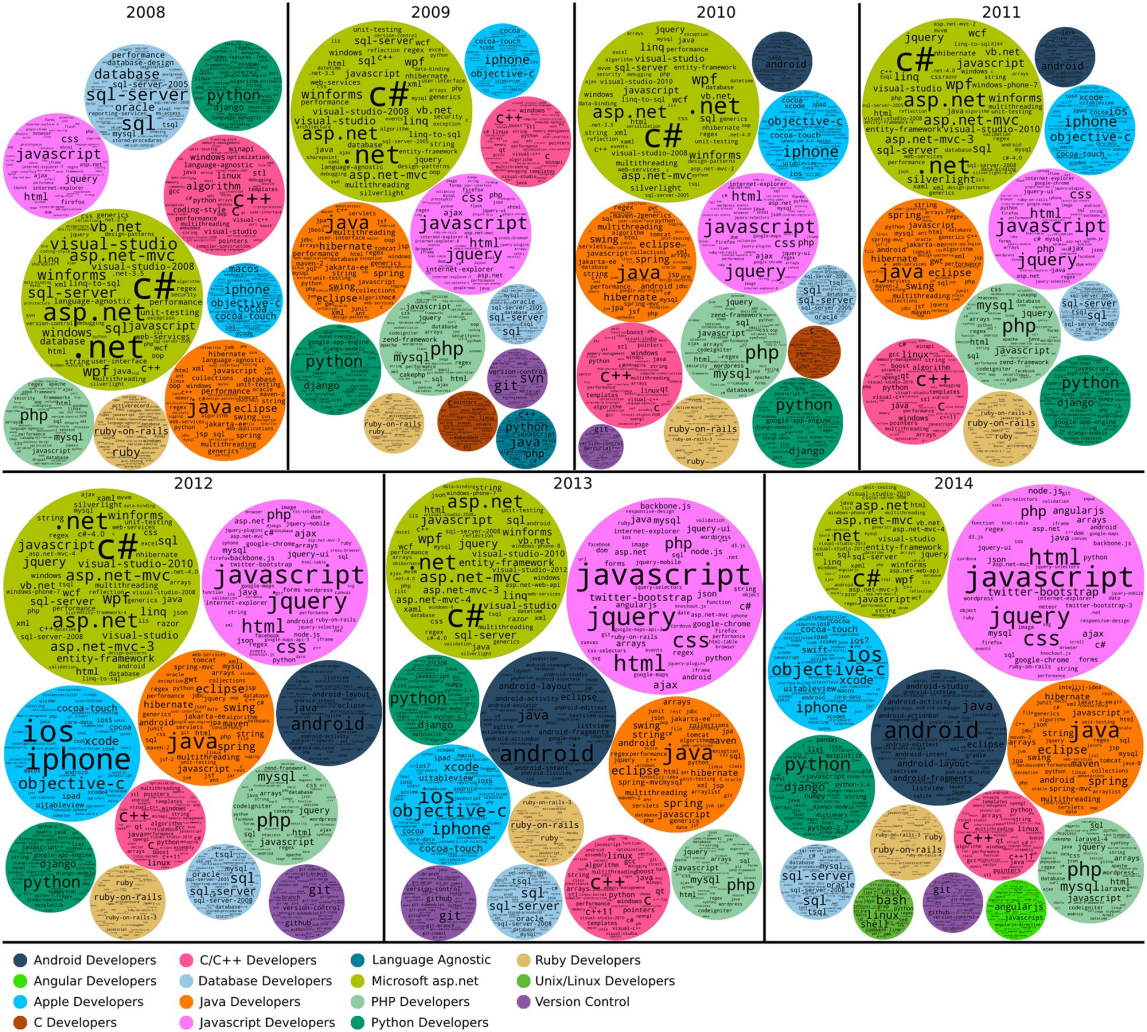
Evolution of the Stack Overflow communities from 2008 to 2014. Each circle shows a tag cloud with the most popular tags in a community identified in a given year. The size of the circles is somewhat proportional to the actual size of the communities, with some adjustments for layout.

**Fig 7:**
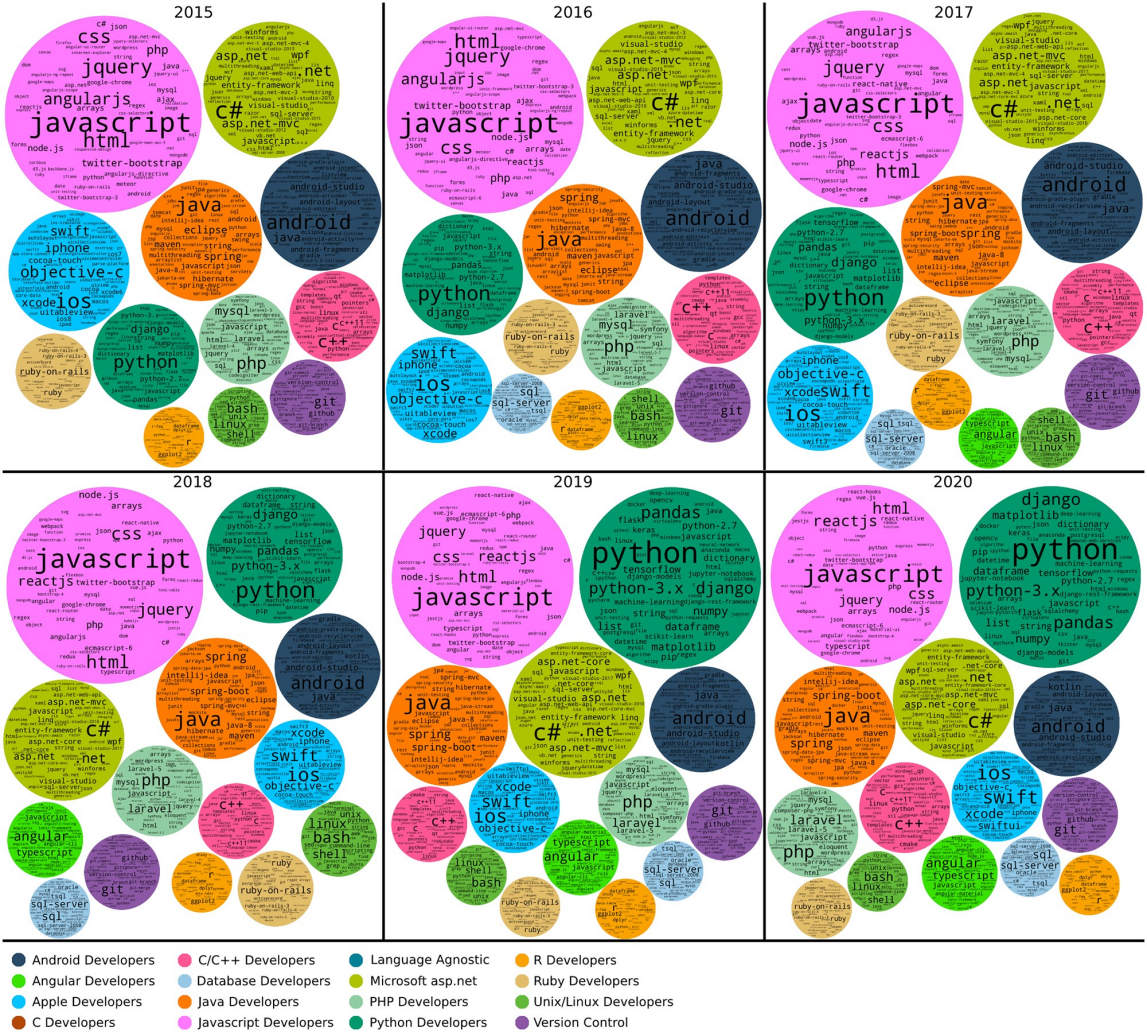
Evolution of the Stack Overflow communities from 2015 to 2020. Each circle shows a tag cloud with the most popular tags in a community identified in a given year. The size of the circles is somewhat proportional to the actual size of the communities, with some adjustments for layout.

To monitor how the developer communities evolve we created a user community graph similar to Fig 5 for every year between 2008 and 2020 inclusive. We then applied the Infomap community detection algorithm to each annual graph. For every detected community, its technological focus or field was determined using the dominant tags used by its members. Figs 6 and 7 present community tag clouds for the major communities found from 2008 (when the Stack Overflow website was launched) until 2020.

Our analysis shows a number of different communities present in each year. In total, we identified 16 user communities for the time period between the August 2008 and December 2020. There were also more communities detected by the InfoMap algorithm but their size was insignificant (less than 1% of the nodes in the complete graph), so these are omitted from further analysis. The time-resolved graphs show the same/similar communities as the time-aggregated graph of Fig 5; the exceptions are the Laravel community, which is not identified in the yearly graphs, and the Testing Developers community, which is only seen in the 2008 graph.

Each community can be characterised by the dominant tags associated with the members of each community. Table 3 shows the dominant tags for the 16 communities found across all graphs that were created, including the time-aggregated graph created with the accumulated data from 2008 to 2020 and the time-resolved annual graphs. Focusing on the users gives a view of the persistence of user populations working with particular technologies, how users acquire score, and how users migrate between technologies. Focusing on the tags associated with each community gives a view of the technology clusters that make up the wider programming and software development ecosystem, including which technologies are typically clustered together in practise, how new technologies arise and are getting adopted, while old technologies fall out of use.

**Table 3:** Community characterisation.

Community Name	Top Tags
Android Developers	android, java, android-layout, android-studio, android-fragments, android-activity, listview, android-intent, xml, eclipse, json, sqlite, android-recyclerview, gradle, android-edittext
Angular Developers	angular, javascript, typescript, html, css, rxjs, angularjs, node.js, ionic-framework, angular-material, ionic2, observable, angular-cli, android, jquery
Apple Developers	ios, objective-c, iphone, xcode, swift, uitableview cocoa-touch, ipad, cocoa, macos, uiview, android, uiviewcontroler, core-data, ios7
C Developers	c, linux, embedded, pointers, gcc, arrays, string, windows, algorithm, assembly, function, unix, struct, performance, memory
C/C++ Developers	c++, c, arrays, c++11, linux, java, python, string, pointers, algorithm, c#, gcc, multithreading, templates, windows
Database Developers	sql-server, mysql, database, tsql, oracle, sql-server-2008 c#, php, stored-procedures, join, java, postgresql, select, sql-server-2012
Java Developers	java, eclipse, spring, maven, intellij-idea, spring-boot, java-8, hibernate, spring-mvc, string, json, javascript, jpa, arrays, amdroid
Javascript Developers	javascript, html, css, jquery, reactjs, node.js, arrays, twitter-bootstrap, ecmascript-6, typescript, redux, express, angularjs, ajax, webpack
Language Agnostic	python, java, php, c++, c, javascript, c#, linux, string, algorithm, arrays, language-agnostic, .net, regex, performance
Microsoft asp.net	c#, .net, asp.net, javascript, asp.net-mvc, sql, sql-server, jquery, html, linq, visual-studio, winforms, entity-framework, css, wpf
PHP Developers	php, laravel, mysql, javascript, laravel-5, html, arrays, jquery, wordpress, eloquent, css, sql, laravel-4, json, string
Python Developers	python, django, pandas, python-3.x, numpy, list, matplotlib, tensorflow, python-2.7, dataframe, string, pip, dictionary, javascript, machine-learning
R Developers	r, dataframe, ggplot2, dplyr, plot, data.table, shiny, list, regex, r-markdown, tidyverse, function, loops, matrix, string
Ruby Developers	ruby-on-rails, ruby, ruby-on-rails-3, javascript, activerecord, jquery, ruby-on-rails-4, rspec, git, html, css, devise, postgresql, heroku, mysql
Unix/Linux Developers	bash, linux, shell, unix, python, awk, c, java, c++, git, regex, grep, javascript, sed, arrays
Version Control	git, github, version-control, git-branch, gitignore, branch, javascript, git-merge, git-commit, git-push, git-stash, ssh, git-submodules, bash, commit

The tags with highest scores for each community are shown. The table displays 16 communities which is the union of the communities detected on the accumulated graph from 2008 until 2020 and all yearly graphs. The ranking is a result of aggregating all tag scores for each year since 2008 for each detected community. A description for each tag can be found on Stack Overflow (https://stackoverflow.com/tags).

### Community persistence over time

To further test whether the apparent persistence of communities shown in Figs 6 and 7 is genuine, we examined user behaviour in terms of whether they stay active in the same community (i.e. continue to ask or answer questions related to their current community), migrate to another community (i.e. begin asking/answering questions in another community), or go inactive (i.e. stop asking/answering questions anywhere on the Stack Overflow platform). For clarity, we define an inactive user as one that stops receiving any additional score from the platform for a given time period; this does not necessarily mean that the user stops using the Stack Overflow website (perhaps as a silent viewer), but that they both stopped creating posts and did not receive votes/responses to earlier posts. This definition views “activity” as a combination of the contribution a user makes to the platform and the acknowledgement (reputation points) they receive from it.

**Fig 8:**
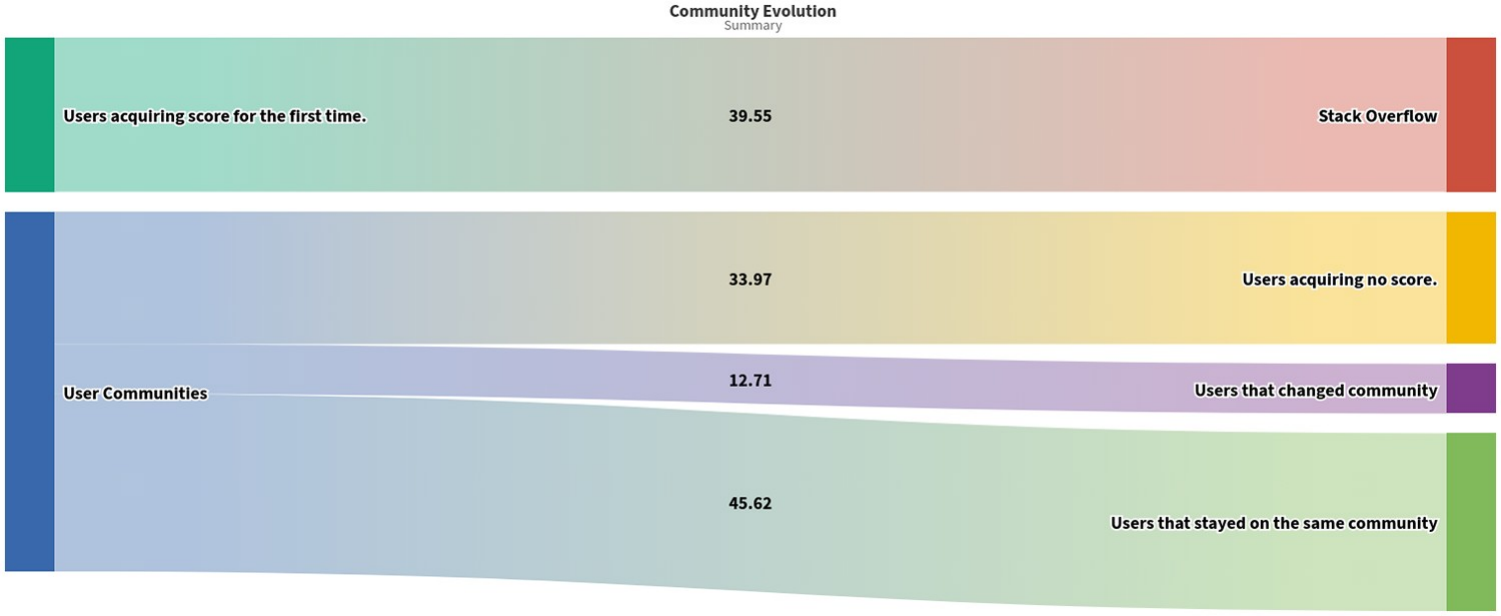
Movement of users in/out/within Stack Overflow. The top node indicates the flow of new users into the platform (users that were not present in the previous year). The second node indicates flows of users that become inactive (i.e. do not acquire any score on the next year), migrate to another community within the platform, or stay in the same community in consecutive years.

Fig 8 shows the average movement rates of users in/out/within the Stack Overflow platform. The top node shows new users moving into existing communities. The bottom node shows users staying active within an existing community, or moving from existing communities to either inactivity or to other communities. On average, ∼45.62% of the users of a community stay in the same community the next year, ∼12.71% migrate to another community, and ∼33.97% of the users go inactive. Also for each year (except 2008) we observed that ∼39.55% of the current users of a community were completely new to the platform.

**Fig 9:**
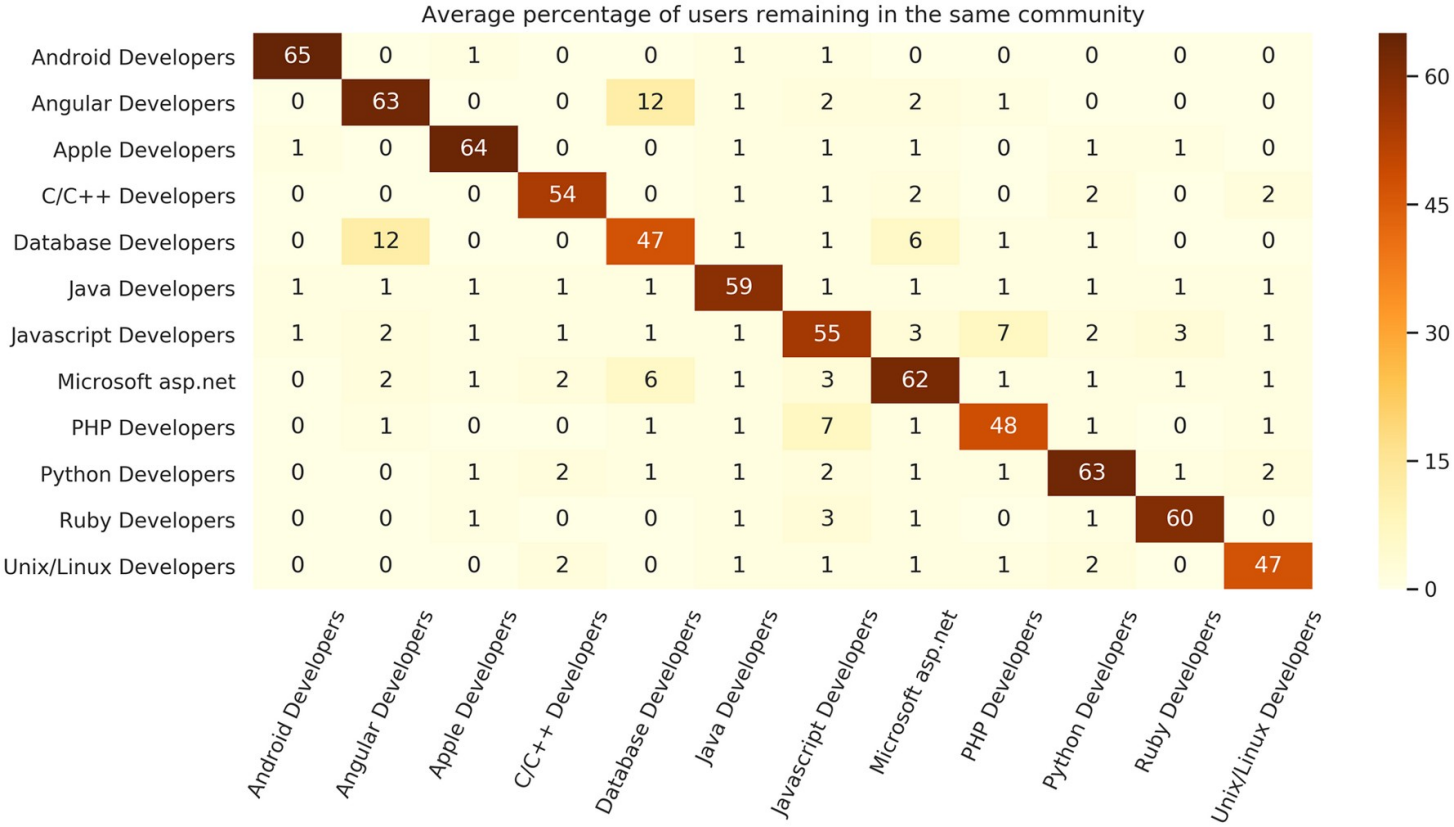
Heat map of year-to-year pairwise similarity of community membership. The heat map displays the average percentage overlap of users belonging to each pair of communities in consecutive years between 2008 and 2020. Diagonal entries indicate self-similarity and community persistence. Off-diagonal entries indicate flows between communities.

Fig 9 shows the average percentage overlap between every pair of communities between consecutive years. The persistence of the user membership of each community is given by entries on the diagonal; the similarity of each community to itself from year to year. The off-diagonal entries indicate the overlap of users between each pair of communities, indicating the flow of users between communities. Between-community flows are typically small.

The identified communities we discuss have large percentage self-similarity and can therefore be seen as persistent from year to year. Note that the percentages shown exclude the (average) 33.97% of users that go inactive in each year, so the percentages displayed in Fig 9 relate to the 56% of users that are retained. Percentages for each line and column do not add up to 100 because of new users entering the platform, and users migrating to minor communities that are not included in Fig 9.

Another way to confirm the consistency of the formed communities is a qualitative comparison of the dominant tags for each community from year to year. Figs 6 and 7 show that the dominant tags for each identified community are coherent between years. Note that consistency does not require the exact same tags to be present in each year, but allows for tags that are associated with the same software field. For example, in the Apple developers community the objective-c tag gets smaller as time passes, while the swift tag is growing; this reflects the decision by Apple to introduce a new programming language for application development in their platforms.

### Community dynamics

**Fig 10:**
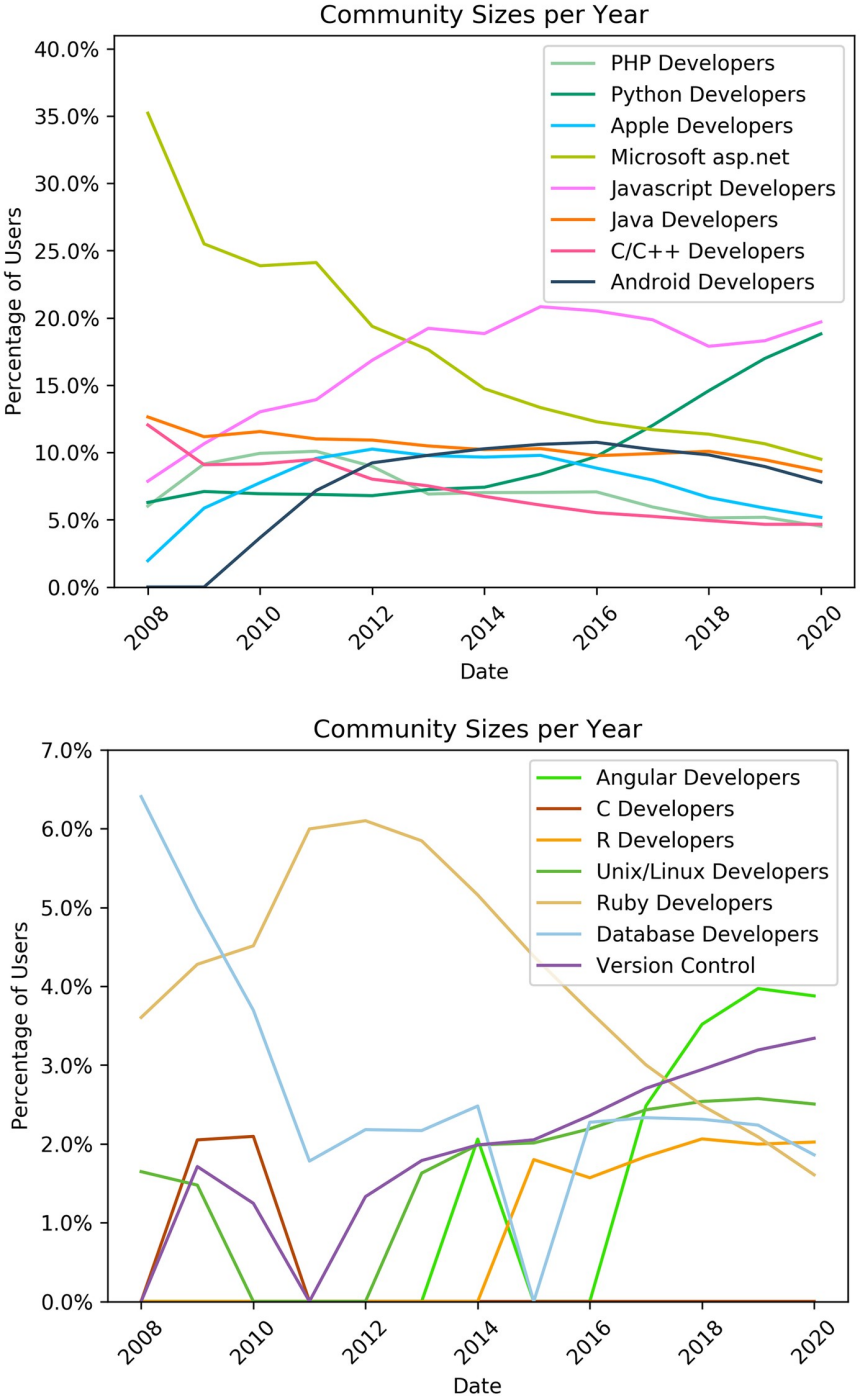
Evolution of community sizes over time. The time series present the percentage of all users on Stack Overflow that each detected community contains in each year from 2008 to 2020. Plots for the 15 largest communities are shown, smaller communities are omitted for clarity.

Early in history of the platform, (e.g. in the 2008 community graph (Fig 6)) we see that only a few communities were formed with the two largest being The Microsoft asp.net and the Java developers communities. Later in the platforms development, many more communities formed and these are more specialised. Fig 10 shows the sizes of the different communities over time. Table 3 shows the major tags in each community. Below we describe the main events for the 11 largest and most persistent communities over time, which are also shown in Fig 10 and characterised based on community graphs and tags.

#### Android developers

This community formed in 2010 and persists until 2020. Users discuss basic components of the Android operating system (layout, fragments, activities), programming languages and developing tools. Top tags include: android, java, android-layout, android-studio, android-fragments, android-activity and listview. Java is the main language for Android development which explains the second-place ranking of this tag. The Android Developers community reached its peak on 2016 where 10% of the total users were part of this community (Fig 10).

#### Apple developers

This community exists since the beginning of the Stack Overflow platform and persists until 2020. Users discuss developing applications on the iOS operating system for mobile devices and the macOS system for personal computers. Dominant tags include ios, objective-c, iphone, xcode, swift, uitableview cocoa-touch and ipad. In Figs 6 and 7, we can observe that the objective-c tag starts to decline after 2016 and the swift tag emerges. The Swift programming language was introduced by Apple in 2014 as a replacement for objective-c in all Apple platforms.

#### C/C++ developers

This community persists since the beggining of the Stack Overflow website. Users primarily discuss the two languages and related tools, as well as other programming languages such as Python or Java to a much lesser extent. Some of the top tags of this community are c++, c, arrays, c++11, linux, java, python, string, pointers, gcc. GCC (GNU Compiler Collection) is the standard (and most well known) compiler for C and C++ on Linux and supports many other languages and platforms as well.

#### Database developers

The Database Developers community formed on 2008 which was also its peak including 6% of the total users. The community almost persists until 2020 with the exception of 2015. Users on this community are discussing about database related topics and technologies such as sql-server, mysql, join and select operations.

#### Java developers

This is another community that was formed in 2008 and persisting until 2020. Users discuss the language itself (versions, libraries), data structures and data types, as well as environments and platforms. Prevalent tags include: java, eclipse, spring, maven, intellij-idea, spring-boot, java-8 and hibernate(Table 3). Eclipse is one of the most well known integrated development environments (IDE) for Java.

#### Javascript developers

This community firs appears in 2008 and grows in size every year until 2015 where its size stabilizes on 20% of the total users of the network. This community is defined by use of the Javascript language and related frameworks (node.js, jquery, ReactJS). Users discuss the language and related tools: javascript, ReactJS, node.js, html, css, jquery, react-native, arrays, typescript, and ecmascript-6.

#### Microsoft asp.net community

The asp.net community is the biggest community from 2008 until 2012. It remains big until 2013 and is still noteworthy until 2020. Some of the top tags of this community, shown on Table 3, are c#, .net, asp.net, javascript, asp.net-mvc, sql, sql-server, and jquery. Those tags show the focus of this community on web development using the .NET ecosystem developed by Microsoft.

#### PHP developers

This community was formed in 2008 and persists until 2020. The pick of its popularity was on 2010 and 2011 including 10% of the network users. The community top tags are php, laravel, mysql, javascript, laravel-5, html, arrays, jquery, and wordpress.

#### Ruby developers

This community exists since 2008 and persists until 2020. The Ruby community is a web development community focused on the Ruby programming language and the associated web framework Ruby-on-Rails. Top tags of the community are ruby-on-rails, ruby, ruby-on-rails-3, javascript, activere-cord, jquery, ruby-on-rails-4 and rspec (Table 3). Community size peaks in 2012 at about 6% of all platform users then declines to about 2% of users in 2020 (Fig 10).

#### Unix/Linux developers

This community concerns the Unix and Linux operating systems. Users discuss the operating systems, associated tools and programming languages. Top tags include: bash, linux, shell, unix, python, awk, c, java, c++, and git. Bash is a command language for the Unix shell; AWK is a domain-specific language used as a data extraction and reporting tool; Git is a version-control system for tracking changes in source code during software development.

#### Python developers

This community formed in 2008 and persists until 2020. Top tags are python, python-3.x, python-2.7, numpy, list, pandas, dictionary, string, arrays, matplotlib. Numpy, Pandas and Matplotlib are Python libraries for data analysis. In 2020, the community reached its peak including almost 20% of the network users.

## Discussion

In this study, we analysed a large amount of data from the Stack Overflow platform to study the communities formed by users around different technologies in the software development and computing industries. By associating users with the tags by which they gathered most ‘reputation’, we formed user profiles that reveal their technology use and expertise. Aggregating these profiles into user-technology graphs (in which two users are connected by the number of tags they share) we were able to detect communities of users. Examining the popular tags within each community allowed us to characterise each community based on its dominant technologies/tools. By repeating the network formation and community detection process, we examined the evolution of the Stack Overflow community over an extended period from 2008 until 2020. To the extent that Stack Overflow is reflective of the wider software and computing industry, trends uncovered in this analysis reflect trends in the digital industries.

Our study reveals a number of insights about the Stack Overflow platform. First, the platform shows strong community structure, with different user populations engaged in discussions around different technology clusters. We were able to detect implicit communities of users specializing in different aspects of software development (e.g. Web Developers, Python Developers, Linux/Unix Developers, and so on). One finding from this is the central position of the World Wide Web and related technologies on software development in the period 2008 to 2020. On average 30% of platform users were members of the Web Developers community in each year in this period, while there were also a number of other web-related communities focused on particular languages or web frameworks (e.g. C#-ASP.NET, Python-Django). Our analysis also explored the movement of active users between communities or in/out of the platform. We found that every year about 39% of the users acquiring score are completely new to the website. Users belonging to a community are likely to either stay in the same community or receive no score for the given year, rather than migrate to a different community within the platform. Finally, we observed an overall pattern whereby the Stack Overflow has changed slowly from a ‘generalised’ discussion, with a small number of larger and more diverse communities, to a more ‘specialised’ mode with a larger number of smaller communities each with a particular focus.

Our findings provide significant insights for professionals working in the computing and software development industries. The communities we identify represent clusters of technologies, and by extension, skills and technical competence. A human resource manager might use this information to tailor job advertising and hiring campaigns, or create appropriate job offers. Evaluation of potential employees would benefit from improved competency profiling processes [25], enabled by using the observed technology clusters to get a better understanding of skills associated with different job roles. A student or a software enthusiast can choose the right technology to start his/her career in software development by examining the size and growth of different communities. Businesses in the tech industries can also benefit from such analysis by being able to better assess a new market; knowing the groupings of technologies and programming languages, and the growth/decline of these groupings, might guide better investment decisions. Software developers can recognize emerging or decaying technologies and adapt to this rapidly evolving industry. Also the grouping of different technologies into communities provides a guide of which groupings of technologies perform better together, providing useful information to system architects.

From an academic perspective, to our knowledge this study presents the first large-scale analysis of community structure within the Stack Overflow platform. Here we showed that weighted networks describing the indirect interactions between users (mediated through their shared usage of the same tags) can be constructed, then interrogated using community detection methods to identify meaningful groups of users. This approach can be expanded in future work to examine (e.g.) clustering of technologies, individual trajectories along a career path, evolution of technological trends, amongst other themes. Stack Overflow is an intriguing example of a complex sociotechnical system [26]. The full user-technology interaction network is a large object with rich structure; the community detection process we used to identify meso-scale patterns within the network is only one kind of analysis that might be applied. While here we tracked network dynamics using a simple one-year time increment, it would be possible to perform a more complex temporal analysis to explore trends at a variety of scales. Stack Overflow is also a rich resource for the study of collaborative work and crowd-production of knowledge. Here we have shown that users tend to stay within a particular segment of the knowledge base. Such findings might help inform efforts to design better systems to manage online collaborative knowledge production. Social scientists can observe how users adopt new skills, learn new ones and abandon skills they already had. They can also observe the life cycle of communities of individuals in the field of software development and monitor how users respond to new technologies or technologies that are getting obscure. Lastly, by investigating how consistent the detected communities are in terms of common users for each year and in terms of software development field they correspond, we demonstrated how community detection methods (infomap in our case) can produce meaningful communities using real life data that were rapidly changing over time.

## Threats to validity

Threats to internal validity concern the community detection algorithm we used (Infomap) which allows each network node (user) to participate on only one community. It is possible that a number of users could be related with more than one communities. Another threat to internal validity is the sparsification of the graphs from 2010 to 2020. We took into account only the top 100 thousand users, based on their reputation, for creating the networks, however we strongly believe that the amount of users is sufficient and the fact that they are the users with the highest reputation helps our method to focus on the authorities of the industry. Lastly another potential threat to validity is the edge creation approach we used to create the user networks. We are not using direct interactions of users such as answers to questions but the similarity of users based on their tags. We strongly believe that this is not a significant threat to validity since although there might be related users that never interacted the tags assigned to them are valid and reveal the actual interests of each user. We are planing on investigating networks created by utilizing direct interactions on the platform on future work.
